# Understanding the associations between receipt of, and interest in, advice from a healthcare professional and quality of life in individuals with a stoma from colorectal cancer: a latent profile analysis

**DOI:** 10.1007/s00520-024-08657-2

**Published:** 2024-06-26

**Authors:** William Goodman, Amy Downing, Matthew Allsop, Julie Munro, Gill Hubbard, Rebecca J Beeken

**Affiliations:** 1 School of Medicine, University of Leeds, Leeds, UK; 2 Department of Nursing, University of the Highlands and Islands, Inverness, UK; 3 School of Health Sciences, University of Dundee, Dundee, UK; 4 Research Department of Behavioural Science and Health, University College London, London, UK

**Keywords:** Quality of life, Advice, Stoma, Colorectal cancer, Physical activity

## Abstract

**Purpose:**

To explore whether profiles derived from self-reported quality of life were associated with receipt of, and interest in, advice from a healthcare professional in people with a stoma.

**Methods:**

Secondary analysis of cross-sectional national survey data from England of 4487 people with a stoma from colorectal cancer. The survey assessed quality of life using various scales, receipt and interest in various forms of advice, and physical activity. A three-step latent profile analysis was conducted to determine the optimum number of profiles. Multinomial regression explored factors associated with profile membership. A series of logistic regression models examined whether profile membership was associated with interest in advice.

**Results:**

Five profiles were identified; ‘consistently good quality of life’, ‘functional issues’, ‘functional and financial issues’, ‘low quality of life’ and ‘supported but struggling’. Individuals in the ‘functional and financial issues’ and ‘low quality of life’ profiles were more likely to have received financial advice compared to the ‘consistently good quality of life’ profile. When compared to the ‘consistently good quality of life’ profile, all other profiles were more likely to report wanting advice across a range of areas, with the strongest associations in the ‘low quality of life’ profile.

**Conclusion:**

Findings indicate that people with a stoma are not a homogenous group in terms of quality of life. Participants in profiles with quality of life concerns report wanting more advice across various categories but findings suggest there is scope to explore how this can be tailored or adapted to specific groups.

**Supplementary Information:**

The online version contains supplementary material available at 10.1007/s00520-024-08657-2.

## Introduction

A stoma is an artificial opening on the abdomen that has been created to divert the flow of faeces or urine [1]. Recent estimates suggest over 175,000 people in the UK live with a stoma, with 13,500 stoma formation surgeries conducted annually [2]. Colorectal cancer is the foremost reason for creation of a bowel stoma [3], with those that have a stoma formed due to cancer having impaired quality of life (QoL) compared to those without a stoma [4]. A systematic review found that stoma complications, a changing body and changes to usual activities can negatively impact QoL, which can be further influenced by demographic and clinical characteristics such as age, gender and the time since cancer treatment [5]. Although QoL increases over time research suggests that it is still lower for those with, compared to those without, a stoma [4]. QoL is increasingly viewed as a key measure to evaluate patients’ recovery from treatment [6] and is incorporated into the NHS long-term strategy to enhance patient care and encourage patient self-management [7]. Therefore, an understanding of how QoL varies across people with a stoma will aid in the development of interventions to increase QoL. Specific groups in greatest need can be targeted by healthcare professionals to improve the relevant aspects of their QOL.

The receipt of advice from healthcare professionals is a common approach to empower patients with the ability to self-manage their condition, adopt healthier behaviours and thus improve their QoL. Behavioural factors such as physical activity can be associated with higher levels of QoL in people with colorectal cancer, and smoking has been associated with lower levels of QoL [8, 9]. A large study of colorectal cancer survivors (*n*=15254) looking at recall of physical activity advice found that receipt of advice from a healthcare professional was associated with reporting meeting physical activity guidelines [10]. A study of research priorities for people with a stoma found that communication with healthcare professionals about living with a stoma was ranked as one of their top priorities [11]. Research has also found that 42% of people living with a stoma had not received physical activity advice and 30% had not received dietary advice but 90% of these individuals wanted to receive this advice [12]. Furthermore, a previous study of people with a stoma from colorectal cancer concluded that ostomy nurses should provide advice to patients in order to help them adjust to specific challenges they may face such as stoma complications [13]. However, not all patients have the same needs, and understanding how to direct and adapt advice could support the delivery of more personalised care in line with the NHS long-term strategy [7].

QoL is a multidimensional construct that can help to capture an individual’s view on their experience of health and allows for the evaluation of interventions designed to improve this [14, 15]. Latent profile analysis (LPA) allows for the identification of subgroups of a sample. This is done by exploring whether there are patterns of responses to certain variables which allow for the exploration of group membership with other variables [16]. We conducted a previous exploratory LPA study which found that people with a stoma can be divided into 4 distinct groups based upon their self-reported QoL [17]. Those in the ‘low quality of life’ profile were more likely to have hernia or bulge, have a newer stoma and were less likely to be physically active whereas those in the ‘some quality of life concerns’ and ‘financial concerns’ profiles were more likely to be younger compared to the ‘consistently good quality of life’ profile. However, in this study, the advice and support that patients needed were inferred from their profile rather than assessed by self-report.

The present study includes this self-report information by utilising population-based data from a survey of colorectal cancer survivors collected in 2013 in England [18]. The primary objective of this study is to use LPA to identify groups of patients, based upon their self-reported QoL, and explore the associations between group membership and advice they received or would like to receive.

## Methods

### Study design

This study is a secondary analysis of a cross-sectional, national survey of colorectal cancer patients. The data were accessed using the COloRECTal cancer data Repository (CORECT-R). The CORECT-R resource, and analyses based upon the data within it, has received approval from the Southwest-Central Bristol research ethics committee (18/SW/0134) and conformed to the ethical guidelines of the Declaration of Helsinki.

### Procedure and participants

Participants that had received a diagnosis of colorectal cancer in 2010 and 2011 and who were still alive as of January 2013 were sent a postal survey with 2 follow-up reminders. Eligible individuals were identified by the National Cancer Registration and Analysis Service and the survey was administered by the National Health Service (NHS) England. A total of 21,802 people responded to the survey; this study is focussed on the 4487 who self-reported that a stoma was present when completing the survey.

### Measures

#### Demographic and clinical characteristics

Sex was measured using a single question and recorded as male or female. Age was from their time of diagnosis. An area-based measure of socioeconomic status was derived using the Indices of Multiple Deprivation [19]. Individuals were assigned to a quintile ranging from 1 (least deprived) to 5 (most deprived) based upon their postcode at diagnosis. Respondents were asked whether they had certain long-standing health problems (e.g. angina pectoris, high blood pressure) which were summed to provide the number of comorbid conditions each individual had, with the maximum being 17. Time since initial treatment was assessed by asking participants whether they were still receiving their initial treatment, or whether they were less than 3 months, between 3 and 12 months, between 1 and 5 years, or more than 5 years since treatment.

#### Behavioural measures

Physical activity was measured by a single item asking how many days in the past week they had been physically active for 30 min or more, in a way that it had raised their heartbeat. Smoking status was recorded as non-smoker, ex-smoker or smoker.

#### Receipt of and interest in advice

Participants were asked whether they had received advice from healthcare professionals across several different categories. They were also asked whether it would have been helpful to have more advice on each of these aspects. These questions came from the Cancer Patient Experience Survey [20] and were important for this study in that they map to key QoL domains. For the purposes of this study, the advice questions were condensed into diet and exercise, any financial information, information for family and friends, physical aspects of living with and beyond cancer and psychological aspects of living with and beyond cancer. Online Resource 1 outlines how these categories were condensed.

#### Quality of life

Several measures of QoL were used in the survey. Participants completed the EuroQol - 5 Dimensions - 5 Levels (EQ-5D-5L) but the visual analogue scale was not included in the questionnaire [21]. The dimensions of self-care (e.g. ‘I have no problems washing or dressing myself’), usual activities (e.g. ‘I have no problems doing my usual activities’) and pain and discomfort (e.g. ‘I have no pain or discomfort’) were used in the present study. Participants completed only the Additional Concerns subscale from the Functional Assessment of Cancer Therapy-Colorectal (FACT-C) scale in order to obtain participants’ views on specific concerns related to colorectal cancer [22](e.g. ‘I like the appearance of my body’). The survey also included the Social Difficulties Inventory [23], which consists of three subscales. The money matters subscale (e.g. ‘Have you had any financial difficulties’) and the self and others subscale (e.g. ‘Have you had any difficulty communicating with those closest to you (e.g. partner, children, parents)’) were used in the present study.

For the EQ-5D-5L, the dimensions were single items and the means were used in the analysis [21]. The dimensions use a Likert scale with 5 options (ranging from no problems to severe problems). Research suggests that Likert responses can be used as continuous measures as long as there are at least 5 levels [24, 25]. The FACT-C Additional Concerns subscale (7 items) score was calculated following the guidance for the scale [22]. The scores range from 0 to 28, with higher scores indicating better QoL. The Social Difficulties Inventory subscales were calculated according to the guidance [23]. The range for the self and others subscale (5 items) was 0-15 and for money matters (5 items) it was 0-13, with higher scores indicating worse QoL. However, to ensure comparability of the scales across the analysis, the scores for the EQ-5D-5L and the Social Difficulties Inventory were reversed so that higher scores indicated better QoL and lower scores indicated worse QoL.

The subscales/dimensions that were used within the present study were selected to allow for comparison with our previous LPA study within this population, which was the 2^nd^ study in a programme of work exploring the factors associated with QoL in people with a stoma [17]. Information on which subscales were selected to be comparable with those of the previous study can be found in Online Resource 2.

### Statistical analysis

Data were analysed using Stata v16.0 and Latent GOLD v6.0.

All variables were summarised descriptively. Whilst the variables of deprivation quintile and time since cancer treatment are summarised descriptively, they were treated as continuous variables in the analyses alongside age, number of comorbidities and days in a week physically active due to having 5 or more categories [26]. The variables with the most missing data were those related to interest in receiving more advice, with missing data at 16.5% for these variables. This was a multi-response question; hence all variables had the same missing data if the question was missed. To account for the missing data in the analysis the maximum likelihood method was used which uses all available data.

The three-step approach to LPA was used for this study. The first step involved identifying the optimum number of profiles. A series of models were run with 1 to 6 (the number of subscales included in the analysis) profiles. These models were assessed on several model fit statistics: Akaike Information Criteria (AIC) and Bayesian Information Criteria (BIC) and entropy. For AIC and BIC, a smaller number indicates a better fit and for entropy a number closer to 1 indicates a better separation between profiles. The size of the smallest profile was also taken into consideration, and if this was below 5% of the sample then this model was not considered. Once the optimum model was selected, participants were assigned to their profile based upon probability scores. To present the QoL scores for each profile graphically, profile scores were standardised from 0 (poor QoL) to 1 (good QoL) for each subscale with the difference between the individual profile subscales and the standardised mean subscale scores calculated. This standardised mean difference was plotted. This approach was taken because of the differing total scores across the subscales used.

A one-way analysis of variance (ANOVA) was conducted to test whether there was an overall difference in the subscales across each profile and post hoc Bonferroni tests were conducted to test for differences in the subscale scores between profiles. A multinomial regression analysis was conducted to explore whether demographic and clinical characteristics, behavioural measures and receipt of advice were associated with profile membership. Overall differences between the profiles were assessed with the Wald Omnibus tests and associations between profiles were assessed with Wald *χ*^2^ pairwise comparison tests, with the Bonferroni correction applied for multiple tests. Finally, a series of logistic regressions was conducted exploring whether profile membership was associated with interest in receiving advice, controlling for clinical and demographic characteristics and whether they had previously received advice.

## Results

### Descriptive statistics

Table 1 provides an overview of the descriptive statistics of the sample. The sample of 4487 people with a stoma from colorectal cancer were predominantly male (60.4%), were between 1 and 5 years from their initial treatment (75.2%), and were physically active for an average of 2.1 days a week (SD=2.4). The majority of participants did not receive advice on any of the reported areas, although 48.6% of participants did report receiving advice on free prescriptions. Participants were most interested in receiving advice on the physical (28.5%) and psychological (24.3%) effects of treatment.

**Table 1:** Descriptive statistics for the sample (*N*=4487)

Variables	*N* (%)
Demographic and clinical characteristics	
Sex	
Female	1589 (35.4)
Male	2712 (60.4)
Missing	186 (4.2)
Age: mean (SD)	71.2 (10.9)
Missing	229 (5.1)
Deprivation quintile	
1, least deprived	917 (20.4)
2	1071 (23.9)
3	1017 (22.7)
4	846 (18.9)
5, most deprived	636 (14.2)
Missing	0
Number of comorbidities: mean (SD)	1.7 (1.5)
Missing	261 (5.8)
Time since cancer treatment	
Still having treatment	185 (4.1)
Less than 3 months since treatment	77 (1.7)
Between 3 and 12 months since treatment	680 (15.2)
Between 1 and 5 years since treatment	3374 (75.2)
More than 5 years since treatment	53 (1.2)
Missing	195 (4.4)
Days in a week physically active: mean (SD)	2.1 (2.4)
Missing	157 (3.5)
Smoking status	
Non-smoker	2231 (49.7)
Ex-smoker	1776 (39.6)
Smoker	393 (8.8)
Missing	87 (1.9)
Receipt and interest in advice	
Advice received: diet, lifestyle and physical activity	
Yes	2211 (49.3)
No	2033 (45.3)
Missing	243 (5.4)
Advice received: financial information	
Yes	2429 (54.1)
No	1815 (40.5)
Missing	243 (5.4)
Advice received: information for family and friends	
Yes	757 (16.9)
No	3487 (77.7)
Missing	243 (5.4)
Advice received: physical effects of treatment	
Yes	1091 (24.3)
No	3153 (70.3)
Missing	243 (5.4)
Advice received: psychological effects of treatment	
Yes	709 (15.8)
No	3535 (78.8)
Missing	243 (5.4)
Interest in advice: diet, lifestyle and physical activity	
Yes	1025 (22.8)
No	2722 (60.7)
Missing	740 (16.5)
Interest in advice: financial information	
Yes	982 (21.9)
No	2765 (61.6)
Missing	740 (16.5)
Interest in advice: information for family and friends	
Yes	564 (12.6)
No	3183 (70.9)
Missing	740 (16.5)
Interest in advice: physical effects of treatment	
Yes	1278 (28.5)
No	2469 (55.0)
Missing	740 (16.5)
Interest in advice: psychological effects of treatment	
Yes	1090 (24.3)
No	2657 (59.2)
Missing	740 (16.5)
Quality of life	
Usual activities: mean (SD) range 1–5	3.8 (1.2)
Missing	61 (1.4)
Self-care: mean (SD) range 1–5	4.5 (0.9)
Missing	34 (0.8)
Self and others: mean (SD) range 0–15	12.9 (2.6)
Missing	486 (10.8)
FACT-C: mean (SD) range 0–28	16.3 (6.2)
Missing	0
Money matters: mean (SD) range 0–13	11.9 (2.2)
Missing	542 (12.1)
Pain and discomfort: mean (SD) range 1–5	4.2 (0.9)
Missing	59 (1.3)

*N* number of participants, *SD* standard deviation

### Latent profile analysis

**Fig. 1:**
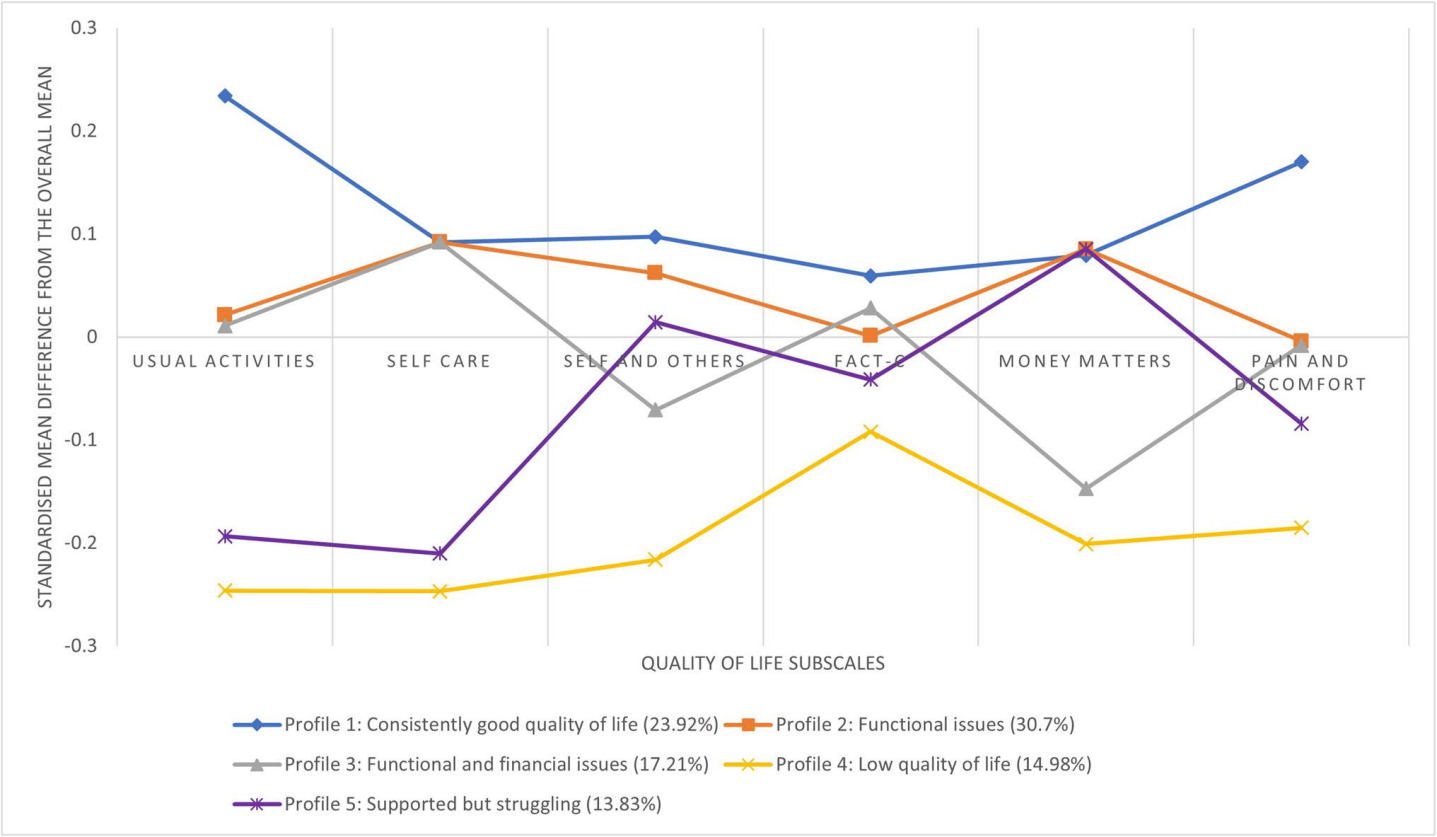
Representation of each of the profiles across the subscales

Based upon the model fit statistics and review of the overall make-up of the profiles the 5-profile model was selected as the optimum model. Model fit statistics for all models conducted can be found in Online Resource 3. Table 2 presents the average scores of the QoL subscales across each of the profiles and Fig. 1 graphically represents the standardised QoL scores for all the profiles.

**Table 2:**
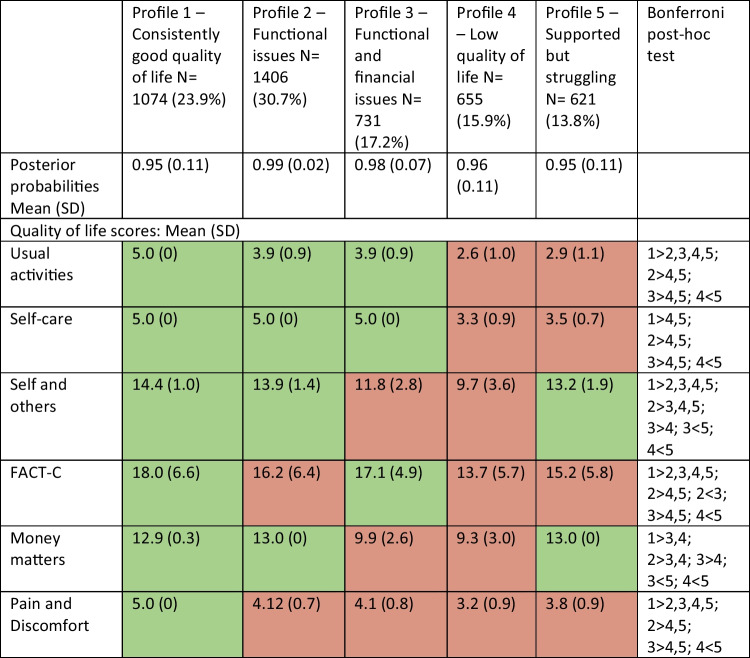
Final profile frequencies and quality of life scores for each subscale

*N *number of participants, *SD *standard deviation, post hoc Bonferroni tests indicate significant differences (*p*<.05) between the profiles, boxes highlighted in green indicate scores above the overall mean and those in red indicate scores below the overall mean

Profile 1 is characterised by higher-than-average scores across all QoL subscales; therefore, this was labelled as the ‘consistently good quality of life’ profile. Profile 2 was characterised as having generally higher-than-average scores on the usual activities, self-care, self and others and money matters subscales but lower-than-average scores on the FACT-C and pain and discomfort subscales; therefore, this was labelled as ‘functional issues’. Profile 3 was characterised by similar scores to profile 2 but with additional problems around money matters and this was labelled as ‘functional and financial issues’. Profile 4 was characterised by lower-than-average scores across all subscales and so was labelled the ‘low quality of life’ profile. Profile 5 had similarly low scores to profile 4 on the usual activities, self-care and FACT-C subscales but high scores on the self and others and money matters subscales and so this was labelled ‘supported but struggling’.

### Association with latent profile membership and interest in advice

Table 3 outlines the descriptive statistics across all the profiles and the Wald Omnibus and *χ*^2^ tests for differences between profiles. Table 4 outlines the results from the multinomial regression with profile 1 ‘consistently good quality of life’ used as the reference group. Individuals that were classified into profiles 2–5 were more likely to have more comorbidities, be closer to when their treatment occurred and be less physically active compared to those in profile 1. Those that were members of profile 3 and 4 were more likely to be younger than those in profile 1 but those in profile 5 were more likely to be older. Those classified into profiles 4 and 5 were also more likely to be smokers than non-smokers and live in a more deprived area than those in profile 1. Those in profiles 3, 4 and 5 were more likely to be male than those in profile 1.

**Table 3:** Descriptive statistics across the profiles

Variable	Profile 1, consistently good quality of life *N*= 1074 (23.9%)	Profile 2, functional issues *N*= 1406 (30.7%)	Profile 3, functional and financial issues *N*= 731 (17.2%)	Profile 4, low quality of life *N*= 655 (15.0%)	Profile 5, supported but struggling *N*= 621 (13.8%)	Wald Omnibus *p* value
*N* (%)						
Demographic and clinical characteristics						
Sex		4		2		<.001
Female	396 (36.9)	548 (39.0)	228 (31.2)	206 (31.5)	211 (34.0)	
Male	628 (58.5)	796 (56.6)	484 (66.2)	421 (64.3)	383 (61.7)	
Deprivation quintile	4	4	4	1-3		<.001
1, least deprived	260 (24.2)	314 (22.3)	145 (19.8)	98 (15.0)	100 (16.1)	
2	276 (25.7)	346 (24.6)	195 (26.7)	127 (19.4)	127 (20.5)	
3	237 (22.1)	330 (23.5)	153 (20.9)	138 (21.1)	159 (25.6)	
4	177 (16.5)	256 (18.2)	138 (18.9)	141 (21.5)	134 (21.6)	
5, most deprived	124 (11.6)	160 (11.4)	100 (13.7)	151 (23.1)	101 (16.3)	
Time since diagnosis	4, 5			1	1	<.001
Still having treatment	25 (2.3)	59 (4.2)	28 (3.8)	41 (6.3)	32 (5.2)	
< 3 months	14 (1.3)	21 (1.5)	18 (2.5)	15 (2.3)	9 (1.5)	
3 to 12 months	133 (12.4)	202 43 (14.4)	151 (20.7)	100 (15.3)	94 (15.1)	
1 to 5 years	861 (80.2)	1056 (75.1)	515 (70.5)	477 (72.8)	465 (74.9)	
> 5 years	10 (0.9)	21 (1.5)	7 (1.0)	7 (1.1)	8 (1.3)	
Smoking status	4			1		0.004
Non-smoker	592 (55.1)	728 (51.8)	360 (49.3)	278 (42.4)	273 (44.0)	
Ex-smoker	394 (36.7)	547 (38.9)	287 (39.3)	276 (42.1)	272 (43.8)	
Smoker	65 (6.1)	103 (7.3)	77 (10.5)	90 (13.7)	58 (9.3)	
Mean (SD)						
Comorbidities	1.2 (0.04)^2-5^	1.7 (0.04)^1, 4, 5^	1.4 (0.1)^1, 4^	2.4 (0.1)^1-3, 5^	2.4 (0.1)^1, 2, 4^	<.001
Age	72.1 (0.3)^3-5^	73.5 (0.3)^3-5^	63.7 (0.4)^1, 2, 4, 5^	68.2 (0.5)^1-3, 5^	76.5 (0.4)^1-4^	<.001
Physical activity	2.9 (0.1)^2-5^	2.1 (0.1)^1, 4, 5^	2.4 (0.1)^1, 4, 5^	1.3 (0.1)^1-3^	1.0 (0.1)^1-3^	<.001
Variable	Profile 1, consistently good quality of life *N*= 1074 (23.9%)	Profile 2, functional issues *N*= 1406 (30.7%)	Profile 3, functional and financial issues *N*= 731 (17.2%)	Profile 4, low quality of life *N*= 655 (15.0%)	Profile 5, supported but struggling *N*= 621 (13.8%)	Wald Omnibus *p* value
*N* (%)						
Receipt of advice						
Advice received: diet, lifestyle and physical activity						0.14
Yes	510 (47.5)	683 (48.6)	403 (55.1)	322 (49.2)	293 (47.2)	
No	500 (46.6)	639 (45.5)	303 (41.5)	304 (46.4)	287 (46.2)	
Advice received: financial information	3, 4	3, 4	1, 2	1, 2		<.001
Yes	499 (46.5)	683 (48.6)	493 (67.4)	420 (64.1)	334 (53.8)	
No	511 (47.6)	639 (45.5)	213 (29.1)	206 (31.5)	246 (39.6)	
Advice received: information for family and friends						0.28
Yes	145 (13.5)	202 (14.4)	155 (21.2)	138 (21.1)	117 (18.8)	
No	865 (80.5)	1120 (79.7)	551 (75.4)	488 (74.5)	463 (74.6)	
Advice received: physical effects of treatment						0.61
Yes	228 (21.2)	317 (22.6)	226 (30.9)	173 (26.4)	147 (23.7)	
No	782 (72.8)	1005 (71.5)	480 (65.7)	453 (69.2)	433 (69.7)	
Advice received: psychological effects of treatment						0.63
Yes	146 (13.6)	194 (13.8)	149 (20.4)	117 (17.9)	103 (16.6)	
No	864 (80.5)	1128 (80.2)	557 (76.2)	509 (77.7)	477 (76.8)	

Superscript numbers relate to Wald *χ*^2^ pairwise comparison tests at *p* < .05 between each class and the class number indicated (1, consistently good quality of life; 2, functional issues; 3, functional and financial issues; 4, low quality of life; 5, supported but struggling); percentages might not add up to 100% due to missing data

**Table 4:** Variables associated with profile membership

	Profile 2, functional issues	Profile 3, functional and financial issues	Profile 4, low quality of life	Profile 5, supported but struggling
Odds ratio (95%CI)				
Sex, male (reference: female)	1.0 (0.8; 1.2)	**1.4 (1.1; 1.8)**	**1.5 (1.2; 1.9)**	**1.4 (1.1; 1.8)**
Age	1.0 (1.0; 1.0)	**0.9 (0.9; 0.9)**	**0.9 (0.9; 1.0)**	**1.0 (1.0; 1.1)**
Deprivation quintile	1.0 (0.9; 1.1)	1.0 (1.0; 1.1)	**1.2 (1.1; 1.3)**	**1.2 (1.1; 1.3)**
Number of comorbidities	**1.3 (1.2; 1.4)**	**1.4 (1.2; 1.5)**	**1.8 (1.7; 2.0)**	**1.6 (1.4; 1.7)**
Time since treatment	**0.8 (0.7; 0.9)**	**0.8 (0.7; 0.9)**	**0.8 (0.7; 0.9)**	**0.7 (0.6; 0.9)**
Days in a week physically active	**0.9 (0.9; 0.9)**	**0.9 (0.9; 1.0)**	**0.8 (0.7; 0.8)**	**0.7 (0.7; 0.8)**
Smoking status (reference: non-smoker)				
Ex-smoker	1.1 (0.9;1.3)	1.2 (1.0; 1.5)	1.2 (0.9; 1.5)	**1.3 (1.1; 1.7)**
Smoker	1.2 (0.9; 1.7)	1.4 (1.0; 2.1)	**2.1 (1.4; 3.0)**	**2.0 (1.3; 3.0)**
Advice received: diet, lifestyle and physical activity, yes (reference: no)	1.1 (0.9; 1.4)	1.0 (0.8; 1.2)	0.8 (0.7; 1.1)	1.0 (0.8; 1.3)
Advice received: financial information, yes (reference: no)	1.0 (0.9; 1.2)	**1.7 (1.3; 2.1)**	**1.7 (1.3; 2.1)**	1.2 (1.0; 1.6)
Advice received: information for family and friends, yes (reference: no)	1.0 (0.8; 1.3)	1.2 (0.9; 1.6)	1.4 (1.0; 1.9)	1.2 (0.9; 1.7)
Advice received: physical effects of treatment, yes (reference: no)	1.2 (0.9; 1.5)	1.2 (0.9; 1.3)	1.1 (0.8; 1.5)	1.2 (0.9; 1.6)
Advice received: psychological effects of treatment, yes (reference: no)	0.9 (0.7; 1.2)	1.0 (0.7; 1.4)	0.9 (0.6; 1.3)	1.2 (0.8; 1.7)

Bold values indicate statistical significance at *p*<.05; where 1.0 is bold, this remains a significant result and is the result of rounding; profile 1 ‘consistently good quality of life’ is used as the reference category for the other profiles

*CI* confidence interval

Across profiles 3 and 4 individuals were more likely to have received financial advice compared to profile 1. But there were no other significant differences observed across the other receipt of advice variables.

Table 5 outlines the results of the logistic regression models exploring interest in receiving advice, with ‘not interested’ as the reference category. These results indicate that having already received advice was associated with less interest in wanting further advice, except for financial information where there was no significant difference. There were also significant results for profile membership being associated with interest in receiving further advice across all advice categories apart from financial information for the ‘Functional issues’ profile. Across all regressions, there were consistent findings that membership of profiles 2, 3, 4 and 5 compared to profile 1 were more likely to be interested in receiving further advice.

**Table 5:** Logistic regression model results exploring interest in receiving advice

	Interest in diet, lifestyle and physical activity advice	Interest in financial information	Interest in advice for family and friends	Interest in advice on the physical effects of treatment	Interest in advice on the psychological effects of treatment
Odds ratio (95%CI)					
Sex, male (reference: female)	0.9 (0.8; 1.1)	1.1 (0.9; 1.3)	1.1 (0.9; 1.4)	**0.8 (0.7; 1.0)**	**0.7 (0.6; 0.9)**
Age	**1.0 (1.0; 1.0)**	**1.0 (1.0; 1.0)**	**1.0 (1.0; 1.0)**	**1.0 (1.0; 1.0)**	**1.0 (1.0; 1.0)**
Deprivation quintile	1.0 (0.9; 1.1)	**1.1 (1.1; 1.2)**	1.0 (0.9; 1.0)	1.0 (0.9; 1.1)	1.0 (0.9; 1.1)
Number of comorbidities	**1.1 (1.0; 1.1)**	**1.1 (1.0; 1.2)**	**1.2 (1.1; 1.3)**	**1.1 (1.0; 1.1)**	**1.1 (1.1; 1.2)**
Time since treatment	1.0 (0.9; 1.2)	0.9 (0.8; 1.0)	1.0 (0.9; 1.1)	1.0 (0.9; 1.1)	1.1 (1.0; 1.2)
Days in a week physically active	1.0 (1.0; 1.0)	1.0 (1.0; 1.1)	1.0 (0.9; 1.0)	1.0 (1.0; 1.0)	1.0 (1.0; 1.0)
Smoking status (reference: non-smoker)					
Ex-smoker	0.9 (0.8; 1.1)	0.9 (0.8; 1.1)	1.0 (0.8; 1.3)	0.9 (0.7; 1.0)	0.8 (0.7; 1.0)
Smoker	1.0 (0.8; 1.3)	1.3 (1.0; 1.8)	1.2 (0.9; 1.7)	1.0 (0.8; 1.3)	0.9 (0.7; 1.2)
Advice received: diet, lifestyle and physical activity, yes (reference: no)	**0.5 (0.4; 0.6)**				
Advice received: financial information, yes (reference: no)		0.9 (0.8; 1.1)			
Advice received: information for family and friends, yes (reference: no)			**0.5 (0.4; 0.7)**		
Advice received: physical effects of treatment, yes (reference: no)				**0.3 (0.2; 0.4)**	
Advice received: psychological effects of treatment, yes (reference: no)					**0.4 (0.3; 0.5)**
Profiles (reference: 1, consistently good quality of life)					
2, functional issues	**1.8 (1.4; 2.3)**	1.2 (0.9; 1.6)	**1.9 (1.3; 2.7)**	**1.8 (1.4; 2.3)**	**2.0 (1.6; 2.6)**
3, functional and financial issues	**2.6 (2.0; 3.4)**	**4.6 (3.5; 6.1)**	**3.9 (2.6; 5.8)**	**2.8 (2.2; 3.6)**	**3.7 (2.8; 4.9)**
4, low quality of life	**2.6 (2.0; 3.5)**	**5.4 (4.0; 7.3)**	**7.0 (4.7; 10.4)**	**4.2 (3.2; 5.5)**	**5.2 (3.8; 6.9)**
5, supported but struggling	**2.0 (1.5; 2.7)**	**1.8 (1.3; 2.5)**	**3.5 (2.3; 5.4)**	**1.7 (1.1; 4.9)**	**2.5 (1.8; 3.4)**

Bold values indicate statistical significance at *p*<.05

*CI* confidence interval

However, the strongest relationships were observed for the ‘low quality of life’ profile across all models, where being a member of this profile was associated with being interested in diet and physical activity advice (OR=2.6, 95%CI: 2.0; 3.5), financial information (OR=5.4, 95%CI: 4.0; 7.3), information for family and friends (OR=7.0, 95%CI: 4.7; 10.4), physical effects of living with and beyond cancer (OR=4.2, 95%CI: 3.2; 5.5) and psychological effects of living with and beyond cancer (OR=5.2, 95%CI: 3.8; 6.9) compared with profile 1.

## Discussion

This study identified 5 distinct profiles of people with a stoma from colorectal cancer based upon their self-reported QoL. ‘Functional issues’ was the largest profile followed by ‘consistently good quality of life’, ‘functional and financial issues’, ‘low quality of life’ and ‘supported but struggling’. Members of the ‘functional and financial issues’ and ‘low quality of life’ profiles were more likely to have received advice on financial matters compared to profile 1, ‘consistently good quality of life’. There were no other differences between the profiles on the advice received. When compared with profile 1, all other profiles were more likely to be interested in receiving advice across all categories. These findings also compliment and confirm some of the findings from our previous LPA study and suggest further avenues for future research and tailoring of interventions [17].

The findings from the present study suggest there is no significant association between receipt of advice and profile membership beyond the receipt of financial information to the ‘low quality of life’ and ‘functional and financial issues’ profiles. This could suggest that health professionals are taking a blanket approach to the advice they provide to patients and not the tailored approach that is recommended and outlined in the NHS long-term strategy [7]. The Association for Stoma Care Nurses (ASCN) guidelines from 2021 indicate a number of areas in which advice and support should be offered to patients but there is no information provided on how to tailor this [27]. Research has suggested that patients can suffer from information overload which can prevent them from taking in information [28]; therefore, it is imperative that patients are not overburdened with possible unnecessary information which they are not interested in receiving.

This study has also identified that those from the profiles with the most QoL concerns, ‘low quality of life’, ‘supported but struggling’ and ‘functional and financial issues’ had the strongest associations with wanting more information for family, friends or carers. This could be due to them relying more on their support or possibly having younger families who could benefit from additional information or advice. A review of research into living with a stoma suggests that those that perceive they have greater social support from their family and friends have less difficulty in adjusting to a stoma than those who perceive they have little social support [29]. Therefore, ensuring that family and friends are included when advice is provided by healthcare professionals to patients may have a beneficial impact on the QoL of the patient.

Members across all profiles were less physically active than those in the ‘consistently good quality of life’ profile. Whilst this is in line with our previous LPA study for the ‘low quality of life’ profile [17], it may suggest that colorectal cancer survivors with a stoma have more functional issues than the wider stoma population. Lower levels of physical activity could also be related to being more likely to have comorbid conditions, or due to them being more likely to be closer to their initial treatment with side effects from certain cancer treatments associated with lower levels of physical activity [30, 31]. However, to determine causality a prospective study would be needed. Furthermore, being a smoker was associated with being a member of the ‘low quality of life’ profile, which previous research suggests is related to lower levels of QoL [9]. Around half of the respondents reported receiving advice on their health behaviour but close to a quarter of people reported being interested in receiving advice on this; therefore, a tailored approach of offering health behaviour advice dependent on individuals’ lifestyle and clinical characteristics may provide more positive outcomes.

This study supports the findings of our previous LPA research in this population [17]. Our previous LPA study identified 2 profiles with financial concerns similar to those identified by the present study, ‘functional and financial issues’ and ‘low quality of life’. The members of these profiles across both studies were likely to be younger [17] which is in line with other research that suggests that the QoL of younger individuals could be more impacted than older individuals [32, 33]. Therefore, it may be that there needs to be more comprehensive advice provided to younger individuals around financial issues. However, this study also identified those from profile 5, ‘supported but struggling’, as wanting more financial advice despite reporting no financial concerns. This could be explained by their reported concerns in carrying out usual activities and self-care and they may be pre-empting a change in their circumstances and want additional financial advice now.

A strength of this study is the large sample size, with the broad range of variables available which amplify our understanding from our previous LPA study amongst this population [17]. Furthermore, we can assess clinically meaningful differences between the profiles across some subscales. For example, for both the FACT-C additional concerns subscale and the subscales for the Social Difficulties Inventory, a change in scores of 2–3 points indicates a clinically meaningful difference [34, 35]. Therefore, apart from the ‘consistently good quality of life’ and ‘functional issues’ profiles, all profiles are different from each other in a clinically significant way. Future studies targeting interventions tailored to each profile could use this approach to look for clinically meaningful improvements in QoL as participants may move between profiles in response to interventions.

There are some limitations associated with this study. Firstly, the measures assessing QoL were generic and were not able to take into account the unique concerns of people with a stoma which would better allow us to identify the physical and mental issues associated with having a stoma and aid in tailoring interventions. This also impacts on the comparability with our previous LPA study which used stoma-related QoL measures [17]. Finally, although these results provide information on the type of advice patients would like to receive which can help in tailoring interventions, this data was collected several years ago and may not be indicative of current stoma care. National guidelines outlined in 2021 [27] have indicated areas for additional support to be offered to patients to facilitate their adjustment to their stoma similar to those outlined in this paper. However, consideration needs to be given to the personalisation of this support to patients which this paper offers advice on.

In conclusion, this study has identified 5 profiles based upon QoL of colorectal cancer survivors with a stoma. The results suggest that people with QoL concerns are not receiving adequate levels of advice across different areas relevant to their well-being, and this is particularly prominent for those who are in the ‘low quality of life’ profile. To improve care for this group of patients, ASCN guidelines on support for patients need to consider how to best to tailor advice to those patients that need it, and how to deliver this advice in a meaningful way.

### Supplementary information

ESM 1 (DOCX 13 kb)

ESM 2 (DOCX 13 kb)

ESM 3 (DOCX 12 kb)

## Data Availability

The data used within this study is available from the UK Colorectal Cancer Intelligence Hub by application.
