# Supplementary material for: Understanding the associations between receipt of, and interest in, advice from a healthcare professional and quality of life in individuals with a stoma from colorectal cancer: a latent profile analysis
Source: Support Care Cancer. 2024 Jun 26;32(7):463. doi: 10.1007/s00520-024-08657-2 (PMC11208265; doi:10.1007/s00520-024-08657-2)
Supplement: Supplementary file 1 — (DOCX 13 kb) [file 520_2024_8657_MOESM1_ESM.docx]

**Original and condensed categories of advice**

| **Original advice categories** | **Condensed advice categories** |
| --- | --- |
| Diet and lifestyle (including smoking) | Diet and exercise |
| Physical activity and exercise |  |
| Financial help or benefits | Any financial information |
| Free prescriptions |  |
| Returning to or staying in work |  |
| Information/advice for family/friends or carers | Information for family and friends |
| Physical aspects of living with and beyond cancer | Physical aspects of living with and beyond cancer |
| Psychological aspects of living with and beyond cancer | Psychological aspects of living with and beyond cancer |
