# Supplementary material for: Understanding the associations between receipt of, and interest in, advice from a healthcare professional and quality of life in individuals with a stoma from colorectal cancer: a latent profile analysis
Source: Support Care Cancer. 2024 Jun 26;32(7):463. doi: 10.1007/s00520-024-08657-2 (PMC11208265; doi:10.1007/s00520-024-08657-2)
Supplement: Supplementary file 2 — (DOCX 13 kb) [file 520_2024_8657_MOESM2_ESM.docx]

**Quality of life subscales used in present study and comparison with those of the previous study**

| Subscales/domains used in present study | Subscales used in previous study (Goodman et al., 2022) |
| --- | --- |
| EQ-5D-5L – Self-Care and Usual Activities | Work/Social Function subscale (e.g. ‘My stoma interferes with my ability to work or attend school’). |
| Social Difficulties Inventory – Self and Others subscale | Sexuality/Body Image subscale (e.g. ‘I am able to share my feelings and concerns about my ostomy with a family member or friend’). |
| FACT-C – Additional Concerns subscale | Stoma Function subscale (e.g. ‘It bothers me if others are aware I have a stoma’). |
| EQ-5D-5L – Pain and Discomfort | Skin Irritation subscale (e.g. ‘I am bothered by skin irritation around the stoma’). |
| Social Difficulties Inventory – Money Matters subscale | Financial Issues subscale (e.g. ‘I have financial concerns regarding my ostomy supplies’). |
