# Supplementary material for: Understanding the associations between receipt of, and interest in, advice from a healthcare professional and quality of life in individuals with a stoma from colorectal cancer: a latent profile analysis
Source: Support Care Cancer. 2024 Jun 26;32(7):463. doi: 10.1007/s00520-024-08657-2 (PMC11208265; doi:10.1007/s00520-024-08657-2)
Supplement: Supplementary file 3 — (DOCX 12 kb) [file 520_2024_8657_MOESM3_ESM.docx]

**Model fit statistics for the latent profile analysis models**

| Number of profiles | AIC | BIC | Entropy | Smallest profile % |
| --- | --- | --- | --- | --- |
| 1 | 102574.1 | 102651.0 | 1.00 | NA |
| 2 | 69555.4 | 69715.6 | 0.96 | 37.7% |
| 3 | 55114.8 | 55358.3 | 0.95 | 20.5% |
| 4 | 46265.5 | 46592.3 | 0.94 | 15.8% |
| 5 | 39354.3 | 39764.5 | 0.95 | 13.8% |
| 6 | 42840.6 | 43334.1 | 0.94 | 2.2% |

Note: AIC= Akaike Information Criteria; BIC= Bayesian Information Criteria.
